# Microbially Influenced Corrosion of Steel in Marine Environments: A Review from Mechanisms to Prevention

**DOI:** 10.3390/microorganisms11092299

**Published:** 2023-09-12

**Authors:** Pan Liu, Haiting Zhang, Yongqiang Fan, Dake Xu

**Affiliations:** 1 Shenyang National Laboratory for Materials Science, Northeastern University, Shenyang 110819, China; 2 College of Life and Health Sciences, Northeastern University, Shenyang 110819, China

**Keywords:** microbially influenced corrosion, biofilms, marine microbiome, MIC prevention

## Abstract

Microbially influenced corrosion (MIC) is a formidable challenge in the marine industry, resulting from intricate interactions among various biochemical reactions and microbial species. Many preventions used to mitigate biocorrosion fail due to ignorance of the MIC mechanisms. This review provides a summary of the current research on microbial corrosion in marine environments, including corrosive microbes and biocorrosion mechanisms. We also summarized current strategies for inhibiting MIC and proposed future research directions for MIC mechanisms and prevention. This review aims to comprehensively understand marine microbial corrosion and contribute to novel strategy developments for biocorrosion control in marine environments.

## 1. Introduction

**Figure 1:**
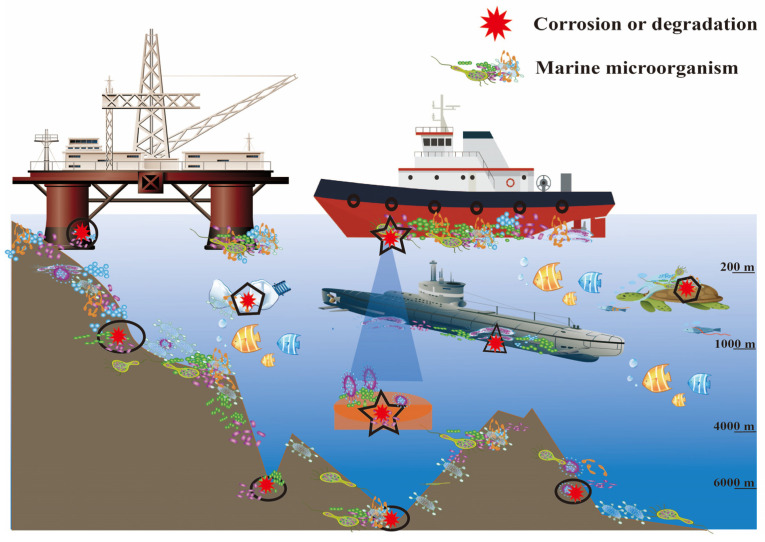
Marine biofilms on different substrates. Microbial coverage is present on various surfaces in marine environments, ranging from the ocean surface to the deep sea. Microbial cells colonize various surfaces such as animals (hexagon), transparent extracellular polymeric particles (TEPs) (pentagon), marine sediments (ellipse), rocks (ellipse), ships (pentagram), submarine (triangle), and mining platforms (circle), leading to the formation of biofilms and the occurrence of pitting corrosion. The occurrence of corrosion is more pronounced in areas with a higher density of microorganisms.

Microbially influenced corrosion (MIC) is widely recognized as corrosion caused by the presence and activities of various microorganisms [1]. Due to its impact on marine steel, MIC imposes significant financial and safety challenges on harbor and port operators globally. Annually, 2.5 trillion US dollars are used for direct corrosion expenses [2,3], 20% of which is attributed to MIC [4], and the data do not include the additional economic costs related to production loss, employee training, research and development, and preventive maintenance. Corrosion-related losses affect critical structural sectors, including offshore oil-gas pipelines, ship hulls, water cooling systems, aviation fuel tanks, sewer systems, and drinking water distribution networks [5] (Figure 1). For example, nuclear power plants have experienced a number of corrosion-related failures since the 1970s, resulting in billions of euros in costs for the industry [6,7]. Consequently, the economic factor is driving the continuous growth of microbial corrosion research [3].

Recently, more and more research related to MIC has yet to be conducted. Though MIC research is a challenging multidisciplinary field, substantial advancements in fundamental research have been made over the past decade. This review summarized recent progress in MIC and microbially influenced corrosion inhibition (MICI) processes and updated our understanding by data mining investigations on microbial corrosion in marine environments.

### Advances in MIC Research in Recent Years

Corrosion in the marine environment is a multifaceted issue influenced by various biological, chemical, and physical factors [8]. As research in this field has been increasing, there is a better understanding of corrosion in the marine environment. Here, we employed bibliometric analysis to summarize the publications on marine MIC in the last 30 years. Bibliometric analysis is a quantitative technique that employs data mining, statistics, and mathematical methods to assess development trends within specific research domains [9]. For instance, Zheng et al. conducted a bibliometric analysis of the literature related to marine environmental corrosion using software such as HistCite, CiteSpace (version 5.7.R1), and VOS viewer (version 1.6.8) [9]. Compared to traditional reviews, this study provides a novel approach to the large-scale quantitative analysis and visualization of marine environmental corrosion.

**Figure 2:**
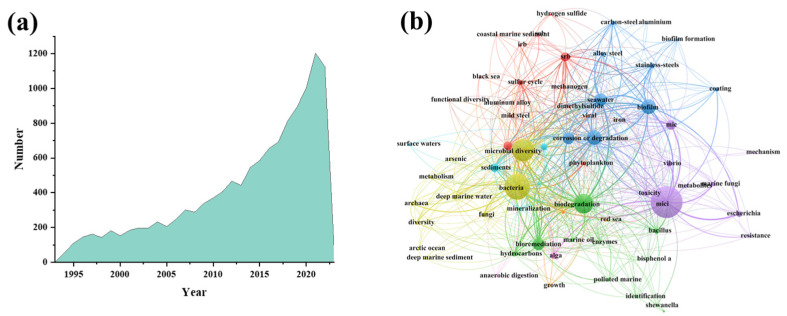
Bibliometric analysis of more than 30 years of research related to marine microbial corrosion. (**a**) Number of papers on marine microbial corrosion research in the last 30 years. (**b**) Different colored lines and circles represent different keywords and their co-occurrence patterns. Co-occurrence network analysis of advances in marine microbial corrosion research.

In this review, we conducted a bibliometric analysis spanning from 1993 to 2023 (Figure 2a), encompassing 12,364 articles, where the specified keywords appearing in the titles, abstracts, and keywords sections were included. We focused on the keywords of the abbreviation “MIC” and vocabulary related to microbiologically influenced corrosion, such as microbiologically influenced corrosion, microbiologically induced corrosion, and microbiologically mediated oxidation. Review studies were excluded, and the analysis results are shown in Figure 2a. During the initial stage before 1997, research on MIC in the marine environment was sporadic as people did not pay the subject much attention. Several events during this period promoted MIC research development. For example, in the mid-20th century, a pipeline rupture in Australia resulted in a substantial oil spill [10,11]. The corrosion of microorganisms garnered attention, marking the beginning of research in the marine MIC field. In the second stage, from 1997 to 2005, researchers began to pay close attention to studying corrosion in the marine environment, resulting in a significant increase in relevant studies. This trend is due to the growing recognition of MIC in the marine environment as a global issue, with governments investing more in this field. During the third stage, after 2005, MIC research of the marine environment experienced exponential growth, primarily due to increasing government investment and the greater number of excellent researchers involved in this field.

To depict keyword co-occurrence, we utilized network visualization as shown in Figure 2b. We retrieved 21,350 keywords, which were clustered into 16 groups. The brightness and intensity of circles and text represent the strength of co-occurrence with other keywords within that cluster. The distance between items reflects their correlation, while the distance between lines represents their correlation. Figure 2b showed that most studies on MIC mainly focused on single-bacteria corrosion, sulfate-reducing bacteria (SRB), nitrogen-reducing bacteria (NRB), and methanogens (MPB) previously related to the circulation of geochemical elements, which cannot fully explain the MIC phenomenon in complex environments. With the development of biological technologies, people can use advanced methods such as in situ technologies, metagenomics, meta-transcriptomics, proteomics, and bioinformatics to study the behavior and mechanism of MIC in real aqueous environments, which contributes to better understanding and addresses environmental problems caused by microorganisms. Researchers can now analyze gene expression and regulation during the process of MIC from a molecular level. These works enabled the identification of marine microbial species that were involved in MIC and promoted the development of MIC mechanisms.

## 2. Microorganisms Involved in Marine MIC

**Figure 3:**
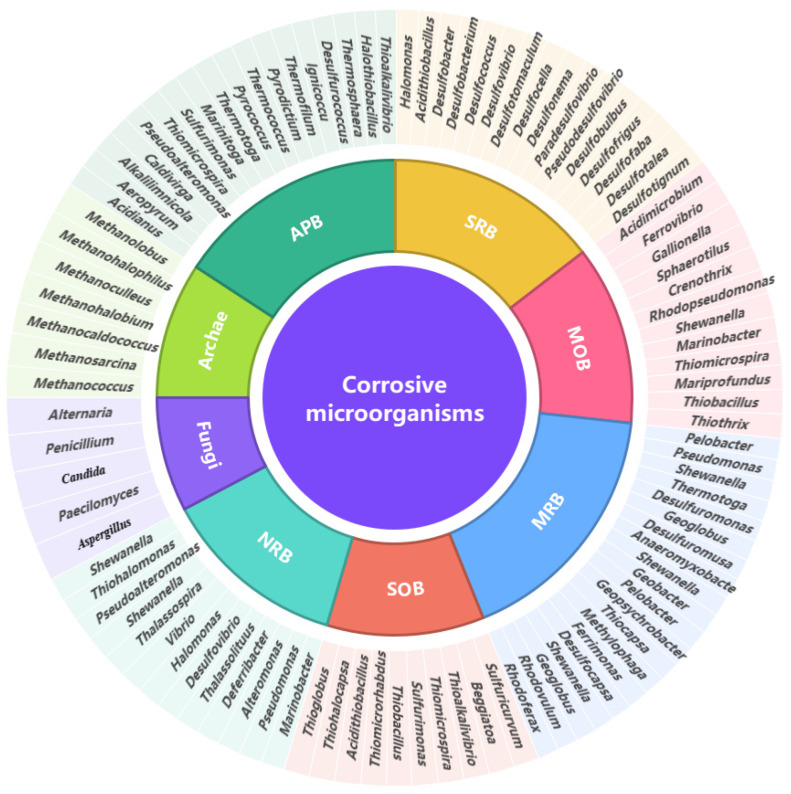
Functional microbes associated with marine MIC. Corrosive microorganisms with different metabolic functions are involved in the marine MIC process.

Corrosive biofilms encompass a diverse assortment of microorganisms, such as bacteria, archaea, and fungi, which contribute to the corrosion process directly or indirectly [12]. The corrosive bacteria involved in the marine MIC process were summarized in Figure 3. The primary types of bacteria associated with steel corrosion vary based on their metabolic modes, mainly including sulfate-reducing bacteria (SRB), sulfur-oxidizing bacteria (SOB), metal-oxidizing bacteria (MOB), metal-reducing bacteria (MRB), as well as microorganisms that secrete organic acids and generate extracellular polymer substances (EPS) [13]. Gaining a comprehensive understanding of the ecological functions of these various microorganisms and the mechanisms by which they influence steel corrosion is essential for the development of effective corrosion prevention strategies.

### 2.1. Marine Sulfate-Reducing Bacteria

Marine sulfate-reducing bacteria (SRB) are anaerobic microorganisms that utilize organic matter as an electron donor during respiration [14]. SRB plays a crucial role in the geochemical element cycle, accounting for more than 50% of sulfate reduction in marine sediments [15]. Additionally, SRB are widely present in marine sediments and have been identified as a leading cause of metal corrosion in the marine environment. This is attributed to the production of acidic by-products during SRB metabolism, which can result in severe corrosion on metal surfaces. Numerous studies have identified many types of marine SRB that inhabit seawater, sediments, and rocks. Currently, more than 60 types of SRB are known to exist in the marine environment [16]. Recent advances in genomics and metabolic pathways have led to new insights into the ecophysiology and distribution of SRB in the marine environment. For instance, studies have demonstrated that SRB is the primary microorganism involved in the long-term seawater immersion process [17]. Prominent SRB species such as *Desulfovibrio* and *Desulfobacter* have been detected in the inner layer of carbon steel, with a significantly higher abundance index than other bacterial species [18].

SRB participates in the corrosion process by producing hydrogen sulfide as the final product of metabolism, which reacts with metal surfaces and forms metal sulfide once the biofilm is established. This reaction releases protons that reduce the pH value of the surrounding environment and produce an acidic microenvironment, further accelerating corrosion [19,20,21]. The metabolic activities of SRB and metal corrosion are interrelated. Corrosion causes the biofilm to thicken and become more complex, altering the electrochemical properties of metal surfaces, and promoting the accumulation of corrosive metabolites, such as hydrogen sulfide and organic acids. These metabolites further stimulate the metabolic activity of SRB, forming a positive feedback loop that accelerates the corrosion rate and leads to extensive damage, making this the main mechanism by which SRB participates in corrosion.

### 2.2. Marine Sulfur-Oxidizing Bacteria

Marine sulfate-oxidizing bacteria (SOB) exhibit contrasting behavior to sulfate-reducing bacteria (SRB), as they are a type of microorganism that oxidizes diverse reduced sulfur species (e.g., hydrogen sulfide, thiosulfate) and elemental sulfur to sulfuric acid under more acidic conditions. A diverse range of SOB is involved in metal corrosion, including both aerobic and anaerobic microorganisms. Among these, sulfide-oxidizing bacteria, particularly those belonging to the *Sulfuricurvum* and *Thiomicrospira* genera, have been extensively researched [22,23]. Other significant SOB groups involved in marine corrosion include *Thioalkalivibrio*, *Sulfurimonas*, and *Thiomicrorhabdus*. Okabe et al. found that SOB could oxidize H_2_S metabolized by SRB, ultimately generating H_2_SO_4_. This process led to a significant decrease in the pH value of the concrete surface from 12.0 to 1.6 after 102 days [24]. Therefore, by leading to acidification of the surrounding environment, SOB can further accelerate metal corrosion.

The corrosion caused by marine SOB can be attributed to two mechanisms of direct chemical reaction and electrochemical corrosion. For the direct chemical reaction, sulfuric acid is produced during the oxidation of sulfur-containing compounds, which can directly corrode metals [25,26]. For instance, when SOB oxidizes sulfides, hydrogen and sulfate ions are generated; these ions react with metals to form metal sulfates, releasing additional hydrogen ions [26]. Electrochemical corrosion involves the formation of local electrochemical cells on the metal surface due to the presence of SOB [27]. During this process, SOB oxidizes sulfur compounds, generating an electron flow through the metal surface and forming cathode and anode regions. As a result, metal ions are gradually lost, forming pits or holes on the metal surface.

### 2.3. Marine Metal-Oxidizing Bacteria

In marine environments, metal-oxidizing bacteria (MOB) use metal as the electron donor for energy metabolism, which causes corrosion of metal materials. Several metal-oxidizing bacteria have been reported to cause the corrosion of metal materials in marine environments, including *Acidimicrobium* and *Mariprofundus*, which participate in the corrosion of iron-based materials by oxidizing ferrous ions. In addition, iron-oxidizing bacteria and manganese-oxidizing bacteria are the two main kinds of metal-oxidizing bacteria. Iron-oxidizing bacteria can oxidize ferrous ions, and produce electronic and acidic metabolites, thus promoting iron oxidation reaction [28]. Manganese-oxidizing bacteria can catalyze the oxidation of divalent soluble Mn (II) to insoluble manganese oxides and accelerate the metal corrosion reaction [29]. These metal-oxidizing bacteria can participate in the corrosion of various metal materials, such as iron, steel, copper, and aluminum [29]. 

In the marine environment, metal-oxidizing bacteria accelerate metal oxidation mainly by their metabolites. For instance, the oxidizing iron–sulfur bacteria (OISSB) participate in metal corrosion processes by oxidizing ferrous ions and sulfide minerals to generate sulfuric acid [30].

### 2.4. Marine Metal-Reducing Bacteria

Marine metal-reducing bacteria (MRB) constitute a diverse group of strictly anaerobic or facultatively anaerobic microorganisms. The representatives of metal-reducing bacteria are iron-reducing and manganese-reducing bacteria. Unlike metal-oxidizing bacteria, these microorganisms utilize metal ions as electron donors for energy metabolism, resulting in the reduction of metals and subsequent corrosion of metal materials. Metal-reducing bacteria, such as *Geobacter* and *Shewanella*, have been extensively investigated for their diverse electron transfer properties [31]. Studies have demonstrated that *Geobacter* has the capability to directly acquire electrons from metal surfaces via an extracellular electron transfer process mediated by its nanowires and outer membrane cytochrome C protein [31,32,33,34].

The mechanisms by which metal-reducing bacteria participate in corrosion can be separated into three main aspects. First, they alter the physicochemical properties of the metal surface by producing a hydrogen oxidation environment through metabolism, leading to the formation of small holes [35]. Second, they reduced insoluble metal oxides (Fe (III) oxides and Cr (IV) oxides) to soluble metal ions to promote corrosion of materials [36,37]. Last, metal-reducing bacteria form a uniform biofilm, which affects the electrochemical process and provides conditions for local corrosion.

### 2.5. Marine Nitrate-Reducing Bacteria

Marine nitrate-reducing bacteria (NRB) are microorganisms that utilize nitrate as an electron acceptor. These bacteria are commonly found in seawater and marine sediments. They are known to cause biocorrosion on marine structures and ship body surfaces, which pose a significant threat to the safety and reliability of marine engineering. *Pseudomonas aeruginosa*, a typical strain of NRB, Chugh et al. discussed the EET corrosion of it in detail [38]. Similarly, Xu et al. reported that the NRB biofilm of *Bacillus licheniformis* developed on carbon steel was more aggressive than that of ordinary desulfurizing bacteria and caused serious corrosion [39].

The kinetics and mechanisms of MIC by NRB involve a complex interplay between biological and electrochemical processes. Biological processes include the attachment and formation of biofilms by NRB on metal surfaces, the reduction of nitrate to nitrite or other nitrogen compounds by NRB, and the production of corrosive metabolites [40]. Electrochemical processes, on the other hand, encompass the formation of galvanic cells between different metal areas or between metal and biofilm, alteration of anodic and cathodic reactions by NRB metabolites, and dissolution of metal ions by acidic or alkaline conditions [1]. These complex interactions can lead to significant corrosion of marine structures and machinery.

### 2.6. Marine Acid-Producing Bacteria

Marine acid-producing bacteria (APB) generate acidic metabolites (organic or inorganic acids) that lead to the corrosion of metallic materials in the marine environment [41]. For example, these aerobic nitrogen-oxidizing bacteria utilize the reducing nitrogen species nitrite (NO^2−^) as an electron donor to produce corrosive nitric acid. Nitrifying bacteria such as *Nitrobacter*, *Nitrococcus*, and *Nitrospira* are included in this category. *Thiobacillus* species can oxidize reduced sulfides such as elemental sulfur, sulfites, and thiosulfates, producing metabolites H_2_SO_3_ or H_2_SO_4_ to erode metals [42]. Acid-producing bacteria of the genus *Desulfurococcus* have also been associated with microbially influenced corrosion (MIC) in marine steelwork. These microorganisms uniquely produce propionic acid as a final product in the electron transport chain.

The metabolic products of marine acid-producing bacteria are involved in the corrosion process by participating in the redox reactions. In contrast to electromicrobial corrosion, these metabolic products are not involved in the corrosion process through the catalytic action of biocatalysts but are directly reduced on the metal surface. Under acidic conditions, the reduction of protons is combined with the oxidation of metal elements to promote the dissolution of metal elements and accelerate the corrosion process [43].

### 2.7. Marine Fungi

Marine fungi are a common group that interacts with living and non-living components of the marine environment. Recent evidence indicates that these fungi are vital in marine food webs and are associated with marine corrosion processes [44]. Marine fungi decompose and utilize various organic materials, thereby promoting the cycling of organic matter and contributing to the stability of marine ecosystems. However, certain species have been found to cause metal corrosion, which reduces the service life and safety of marine facilities [29,45]. The corrosion is mainly due to the secretion of acidic substances during fungal metabolism and the production of metal ions [46]. As an example, genera such as *Aspergillus*, *Candida*, and *Paecilomyces* produce acidic metabolites that accelerate the corrosion of metals such as iron and copper. Moreover, some marine fungi secrete exogenous enzymes such as proteases and cellulases that accelerate metal corrosion by producing additional acidic substances through the degradation of organic matter and proteins on the surface of marine facilities.

The corrosion caused by marine fungi is mainly due to the secretion of acidic metabolites during fungal metabolism and the production of metal ions. For example, species such as *Aspergillus* produce acidic metabolites that accelerate the corrosion of metals such as iron and copper, reducing the durability of marine structures [47]. Additionally, some marine fungi produce exogenous enzymes such as proteases and cellulases that exacerbate metal corrosion [48,49]. These enzymes promote the breakdown of organic matter and proteins on the surface of marine facilities, leading to the production of additional acidic substances and accelerating the progression of corrosion.

### 2.8. Marine Archaea

In recent years, there has been an intensified research focus on the archaeal groups involved in microbial corrosion, particularly in the marine environment. This is due to their significant impact on the corrosion of marine structures, such as pipelines, ships, and oil rigs, which can result in economic losses and environmental hazards [50]. Methanogens and sulfur oxidizers are the primary archaeal groups involved in marine microbial corrosion, as studies have shown [18,51,52,53,54]. Methanogens produce methane by reducing carbon dioxide or organic matter in anoxic sediments or water [55]. This can lead to the formation of cathodic areas and consequently promote the corrosion of metal structures. Conversely, sulfur oxidizers use sulfur compounds, such as sulfide and elemental sulfur, as electron donors for energy metabolism and produce sulfuric acid, which can cause metal corrosion.

Studies have shown that archaea can modify the physicochemical properties of the material surface, such as pH, potential, dissolved oxygen concentration, and ion concentration, by forming biofilms or rust nodules [56,57,58,59]. These alterations influence the electrochemical corrosion process of the material. Additionally, archaea can cause local corrosion or damage to the metal via direct or indirect chemical reactions with the metal through their metabolic products or by-products [58]. For instance, methanogenic archaea (MA) can reduce carbon dioxide to methane using hydrogen or metal as electron donors [59,60]. Sulfate-reducing archaea (SRA) can oxidize organic matter or hydrogen to hydrogen sulfide using sulfate as an electron acceptor [60]. Sulfur-oxidizing archaea (SOA) can use oxygen or nitrate as electron acceptors to oxidize sulfur or sulfide to sulfate [61]. These metabolic processes modify the material surface’s electron density and pH, promoting the metal’s dissolution or pitting corrosion. Moreover, methane, hydrogen sulfide, and sulfate can react with the metal, causing stress corrosion cracking or passivation layer destruction.

## 3. Mechanisms of MIC in the Marine Environment

From a microbial perspective, the selective attachment of microorganisms to metal materials indicates their quest for survival. Microorganisms view metal materials as energy “providers” and attach to them in order to survive [8]. Researchers have found that alloy elements and surface microstructure affect the rate of steel’s MIC in different ways. For example, both stainless steel and carbon steel are iron-based materials, but their surface MICs differ significantly. The passivation film on stainless steel slows down the corrosion process of microorganisms; thus, the corrosion rate of stainless steel is much lower than that of carbon steel in seawater [62]. Although the corrosion mechanisms of stainless steel, alloy steel, and iron-containing steel are different, there are similarities in the microbial destruction processes; therefore, we aimed to summarize the common corrosion mechanisms.

**Figure 4:**
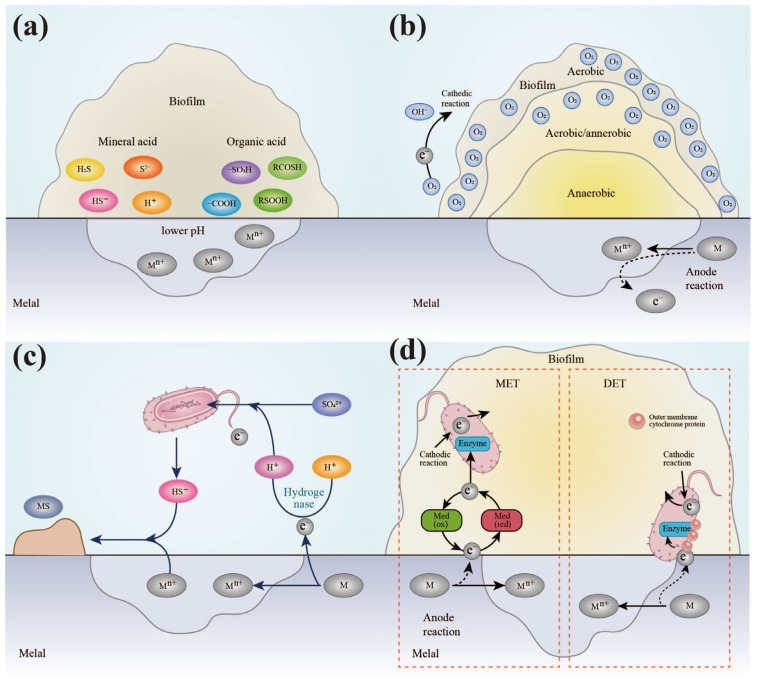
Schematic diagram of corrosion mechanism of marine microorganisms. In marine environments, microorganisms are involved in the corrosion process in the form of biofilms, and several corrosion mechanisms have been illustrated: (**a**) oxygen concentration difference cell; (**b**) corrosion product; (**c**) biocatalytic cathodic sulfate reaction; (**d**) extracellular electron transfer.

Although the role of microorganisms in the corrosion process has been comprehended for over a century, potential hazards of MIC have largely been overlooked. Previously, most work focused on checking the corrosion behavior of microorganisms, but molecular mechanisms of microbial corrosion remain largely unknown. Classic theories, such as the oxygen concentration difference cell theory (Figure 4a) and the corrosion product (Figure 4b) hypothesis, suggested that microbes are not directly involved in the corrosion process. However, these theories are limited and require more evidence in order to fully explain the corrosion process. In 2009, Gu et al. proposed the biocatalytic cathodic sulfate reaction (BCSR) hypothesis based on bioenergetics and bioelectrochemistry, marking a significant step forward in MIC research (Figure 4c) [63]. This hypothesis suggests microbial corrosion is an active process in which microorganisms extract electrons directly from the metal surface. Subsequently, numerous studies have been published to elucidate, support, and refine the BCSR theory. For example, Xu’s starvation tests demonstrated that sulfate-reducing bacteria can use iron as an electron donor to provide energy for maintenance even without a carbon supply or electrons from external sources, which eventually resulted in metal corrosion [64,65]. Xu et al. demonstrated that sulfur reduction occurs within SRB, whereas the oxidation of insoluble iron takes place extracellularly. Therefore, electrons generated from iron oxidation must first travel through an electron transfer chain to the cell wall to participate in sulfate reduction. Thus, a novel theory called extracellular electron transfer (EET) is presented to explain how electrons are transported extracellularly to erode the metal, which is widely accepted as one of the molecular mechanisms for MIC.

Figure 4d illustrates two types of EET processes between microorganisms and metal surfaces: direct electron transfer (DET) and mediated electron transfer (MET) [66]. Direct electron transfer refers to the short-distance transfer of electrons through cytochrome proteins on the outer membrane surface or long-distance transfer via conductive biological nanowires [67,68,69]. An example of direct electron transfer is seen in the sulfate-reducing bacteria *Desulfovibrio ferrophilus* IS5, which can acquire electrons from solid sulfides through an OMCs-like cytochrome-dominated pathway to respire and survive under starvation conditions [70,71]. Tang et al. demonstrated that direct electron transfer is an important mechanism for regulating microbial corrosion [72]. They showed that gene knockouts related to direct electron transfer in *Geobacter sulreducens* and *Geobacter metallireducens* can lead to direct iron-to-microbial electron transfer, which can corrode stainless steel. Similarly, Jin et al. proved that e-pili accelerated the electron transfer between Geobacter and metal and promoted the microbial corrosion process [70]. Indirect electron transfer, on the other hand, occurs when microorganisms secrete soluble electron transfer mediators such as phenazines, flavins, quinones, humic acids, anthraquinone-2,6-disulfonate, and neutral red to absorb electrons from the metal surface [73]. These mediators transfer the electrons to the cell membrane and release them through c-type cytochrome. Riboflavin and FAD have been found to accelerate the corrosion of 304 stainless steel and carbon steel by *Desulfovibrio vulgaris* [74]. Similarly, researchers have utilized cross-fusion molecular biology techniques to determine the role of phenazine compounds in regulating the corrosion of marine *P. aeruginosa* bacteria at the gene level [75]. Specifically, the use of gene knockout technology has revealed that phenazine compounds, encoded by *phzH* and other genes, act as extracellular electron carriers [76]. This advancement promotes the study of the microbial corrosion mechanism to a molecular level, providing a theoretical foundation for investigating the corrosion mechanism of electroactive microorganisms. The findings confirm the crucial role of electron transfer in microbial-mediated metal corrosion and provide a comprehensive understanding of the mechanism of microbial regulation of metal corrosion.

## 4. Multi-Species Biofilms: Accelerating Corrosion through Metabolic Interactions and EET

**Figure 5:**
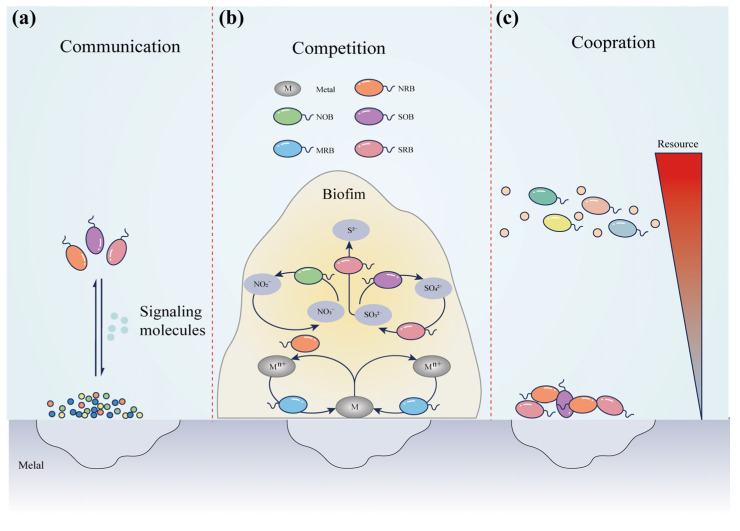
Schematic diagram of corrosion mechanisms of marine microbiome. In marine environments, microbiomes form biofilms on the surface of metal materials, participating in the corrosion process through interspecific relationships: (**a**) communication; (**b**) competition; (**c**) cooperation.

The corrosion of materials by multiple microbial biofilms is notably detrimental to the natural environment, especially in the marine environment. Mixed microbial communities can form complex biofilm structures that create localized environments with low oxygen concentrations, high nutrient levels, and corrosive media [77]. Different species of microorganisms within a biofilm can interact with each other, leading to biochemical reactions that intensify corrosion on the metal surface. The combined synergistic and competitive interaction between multiple microorganisms usually leads to more severe MIC than that caused by each single microorganism (Figure 5). For example, sulfate-reducing and sulfur-oxidizing bacteria can work in tandem to degrade metallic materials, in which the cooperation between these two bacteria species would result in the production of organic acids, lower the pH of the environment, and accelerate the carbon steel corrosion process [78]. Recent studies have demonstrated that the synergistic effects of different types of microorganisms can hasten the corrosion process [79]. Nevertheless, this phenomenon has mainly been established between two or three types of microorganisms, and research on the corrosion mechanisms of more than five types of microorganisms remains scarce. Therefore, further investigation is necessary in order to examine the corrosion mechanisms of larger microbial communities.

Communication. Microbial community behavior regulation mechanisms significantly influence the growth, structure, and function of biofilms. As shown in Figure 5a, to regulate their collective behavior, bacteria employ a cell-to-cell communication system known as “quorum sensing (QS)” [80]. Quorum sensing is defined as an environmental sensing system that enables bacteria to monitor their population density and coordinate group behavior through a wide range of chemical signaling molecules [80,81]. One classic example of adverse biofilm effects is MIC. Additionally, QS mechanisms have been found to affect biofilm formation positively and negatively. Therefore, QS plays a crucial role in mediating MIC by modulating biofilm formation by the microbiome. The relationship between quorum sensing and microbial corrosion was investigated by Umarevathi et al., whose results demonstrated that researchers can effectively inhibit the biocorrosion of metals by QS inhibitors [81]. For instance, Methyl eugenol inhibited microbiome corrosion of 316L stainless steel [82].

Cooperation and competition. Microbial cooperation and competition interactions are essential in accelerating metal corrosion (Figure 5b,c) [58]. Videla and Herrera (2005) pointed out that competition between different types of microorganisms can exacerbate corrosion, as it can lead to the establishment of more aggressive microbial communities [83]. Recent research has indicated that microbes can obtain electrons through electron shuttles or nanowires to support their metabolic processes, potentially resulting in the corrosion of metals [84,85,86]. As mentioned, the transfer of electrons can occur across different species, thereby allowing microbial communities to collaborate in corroding objects in marine environments. Iron-reducing bacteria and iron-oxidizing bacteria are two examples of species that engage in electron transfer and can contribute to metal corrosion. Iron-oxidizing bacteria use oxygen as an energy source, generating Fe (III) that can be transferred to iron-reducing bacteria [87]. This exchange of electrons can encourage the formation of anodic regions on metal surfaces, accelerating metal corrosion. Likewise, iron-oxidizing bacteria and sulfur-reducing bacteria can also collaborate in electron transfer and contribute to metal corrosion. For example, IOB forms biofilm on the surface of the material, creating a micro-anaerobic environment for the growth of SRB, thereby promoting the corrosion of the material [88]. Microbial competition occurs when different species compete for resources such as nutrients, oxygen, and space, producing corrosive agents such as organic acids and hydrogen sulfide (Figure 5c) [28]. For example, sulfate-reducing and acid-producing bacteria compete for nutrients and produce organic acids that speed up metal corrosion in marine environments [89].

Therefore, interspecies relationships of microorganisms in the marine environment have a catalytic effect on metal corrosion. Comprehensive research is necessary in order to examine the corrosion mechanisms of multiple microorganisms to better understand how MIC occurs under natural conditions. These mechanisms are still under investigation, and researchers are exploring the potential influence of microbial communities on infrastructure and the marine environment.

The salinity, pH, temperature, dissolved solids, and biological factors in seawater all affect the corrosion process. The synergistic action of these biological and abiological factors alters the electrochemical process and accelerates the corrosion process caused by microorganisms [90]. For example, the corrosion rate of 6061 aluminum alloy by *Escherichia coli* increases with the increase in seawater salinity [62]. These salinities not only maintain the optimal environment for microbial growth but are also involved in the corrosion damage process. Similarly, chloride ions can destabilize the oxide film, and SRB utilizes sulfates in seawater to generate organic and inorganic precipitates, thereby destroying the oxide film [91].

## 5. Marine MIC Inhibition

**Figure 6:**
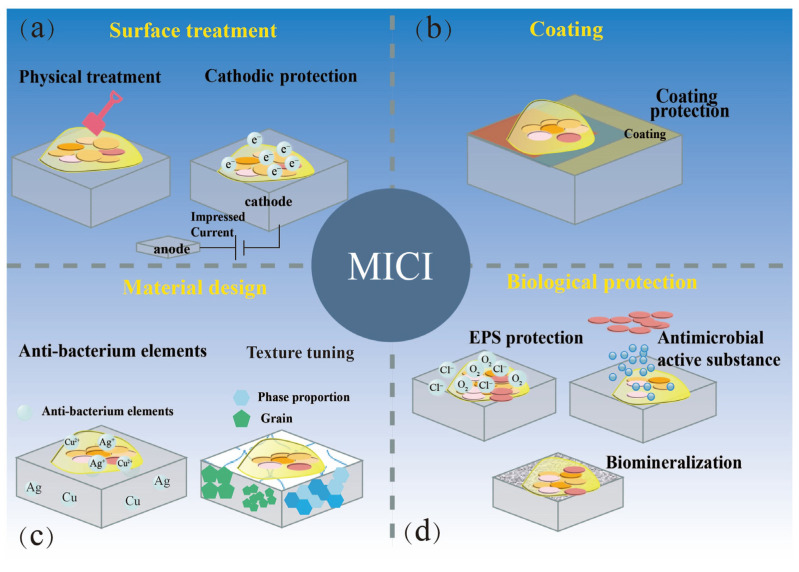
Inhibition of MIC in the marine environment. (**a**) Surface treatment: physical treatment and cathodic protection; (**b**) coating; (**c**) material design: adding antibacterial elements and texture tuning; (**d**) biological protection: EPS protection, antimicrobial active substance, and biomineralization.

A key impetus behind the investigation of MIC mechanisms lies in the pursuit of developing enhanced mitigation strategies. At present, MIC inhibition strategies encompass several general categories, such as surface treatment, coating, material design, and biological protection (Figure 6).

### 5.1. Surface Treatment

**Physical treatment**. MIC is primarily caused by the adhesion and growth of marine microorganisms on the material surface [12]. The formation of biofilms plays a crucial role in inducing these phenomena. In the marine environment, periodic cleaning is a frequently employed method by which to inhibit MIC [92]. However, this method necessitates harsh operations and the cleaning effect in micro-areas is not conspicuous [92]. Thus, it is essential to acknowledge the difficulty of removing the accumulated biofilms [93].

**Cathodic protection**. Cathodic protection (CP) has been widely used in marine engineering, shipbuilding, and other fields, which can significantly extend the service life of metal materials and inhibit the occurrence of MIC [94,95,96]. Some studies have investigated the effectiveness of CP in suppressing MIC [94,95,96]. Cathodic protection is an electrochemical method used to safeguard metal structures against corrosion by applying an external current [97,98]. By making the metal surface act as a cathode, this process effectively hinders the migration of electrons during corrosion, leading to a reduction in the corrosion rate. The cathodic potential created by cathodic protection is unfavorable for microbial growth and biofilm formation, effectively inhibiting the development of MIC. In addition, cathodic protection may enhance metal corrosion sometimes. For example, the application of cathodic protection against SRB or *vibrio* bacteria may demand an increase in cathodic current, and a high cathodic current will increase the possibility of failure in corrosion protection [13]. Due to this reason, the use of this technology must be approached with great caution, and a comprehensive analysis of the biological and chemical factors involved in the entire system is necessary.

### 5.2. Coating

Thus far, various techniques have been explored to prevent corrosion, and coating technology has emerged as a particularly effective method by physically preventing corrosive agents from infiltrating. Anti-corrosion coatings have been developed using a variety of materials, including organic, inorganic, and mixed coatings [99,100].

**Organic coatings.** Organic coatings are extensively used due to their remarkable adhesion, flexibility, and corrosion resistance [101,102]. These coatings can be applied through spraying, brushing, or rolling, and are typically utilized in marine applications, such as hulls, pipes, and offshore structures [102]. Among the various types of organic coatings, epoxy coatings are the most widely used for their anticorrosive properties in the marine environment [101]. As a common polymer, epoxy coatings can form a hard and durable layer that provides adequate protection to metals and prevents microbial corrosion. Other polymer-based coatings such as polyurethane, polyethylene, and polypropylene have also been employed. Polyurethane-based coatings have been shown to possess excellent resistance to marine biofouling and biological corrosion [103]. These coatings serve to create a smooth surface and inhibit the attachment of microorganisms. Conversely, polyethylene and polypropylene coatings have exhibited good resistance to mechanical wear and abrasion [103]. Nonetheless, despite the impressive anticorrosion performance of current organic coatings, they remain susceptible to degradation in the presence of complex flora.

**Inorganic coatings.** Inorganic coatings, also known as metallic coatings, such as metal oxides, nitrides, and carbides, have garnered significant attention due to their high resistance to corrosion and stability in extreme environments [104]. These coatings are typically made up of metals, including copper, zinc, nickel, and aluminum, which have a lethal impact on microorganisms. Among these, copper-based coatings are commonly utilized due to their excellent antifouling properties [105]. They prevent the growth of microorganisms by releasing copper ions [106]. Zinc-based coatings also exhibit promising antifouling characteristics, while nickel-based coatings have demonstrated effectiveness against sulfate-reducing bacteria [107]. Aluminum and its alloys offer exceptional corrosion resistance and can be utilized in various marine applications. 

**Mixed coatings.** Mixed coatings have emerged as a promising method with which to enhance the protection against microbial corrosion by combining the benefits of organic and inorganic coatings. These coatings are composed of two or more materials and have shown improved corrosion resistance and mechanical properties [108]. Of particular interest is the polymer-ceramic blend, which exhibits unique properties such as self-healing and thermal stability, and has been the subject of significant research [109]. In addition to exploring specific coating materials, various strategies have been developed to enhance coating performance against microbial corrosion, including surface modification methods such as plasma treatment [110], micro-arc oxidation [111,112], and sol–gel coating [99,100]. These techniques improve coating adhesion and durability. Another strategy is to add antibacterial agents such as biocides, enzymes, and peptides into the coatings, which can inhibit microbial growth and prevent biofilm formation on the coating surface [113].

### 5.3. Material Design

In microbial corrosion research, establishing a microbe–metal relationship has always been a major challenge. Selective attachment to metal surfaces is a survival mechanism employed by bacterial communities, with the materials often regarded as energy providers [8]. Additionally, alloying elements and surface microstructure impact biofilm formation [114]. Although stainless steel and carbon steel are iron-based materials, they exhibit different surface MIC behaviors. The formation of a passivation film between biofilm and substrate is closely related to Cr and Mo on stainless steel, leading to a lower corrosion rate compared to carbon steel in seawater [8]. Consequently, researchers aim to enhance corrosion resistance through material design and preparation.

**Adding antibacterial elements and texture tuning**. The use of various alloying elements to impart antimicrobial properties is a reliable and effective technique for enhancing the corrosion resistance of steel. Copper, silver, and chromium are examples of metals that can impede bacterial adhesion [115]. Typically, there are three primary antibacterial mechanisms of metal elements: First, negatively charged substances on the membrane and cell walls of bacteria interact with positively charged metal ions, leading to bacterial death by disrupting their living environment [116,117]. Second, metal ions bind to protein groups in bacteria, rendering enzymes inactive and denaturing membrane proteins, thereby hindering bacterial division and proliferation [118]. Finally, metal ions can catalyze the generation of reactive oxygen species (ROS) from oxygen in water and air, which can inflict oxidative damage on vital bacterial components such as proteins, nucleic acids, and lipids, leading to bacterial death [119]. For instance, 317L-Cu stainless steel (SS) exhibited considerable antibacterial activity against Staphylococcus aureus, reducing sessile cell counts by 98.3% compared to 317L SS [120]. In *Escherichia coli*-containing medium, 304L-Cu SS had shallower pitting depth and lower weight loss than 304SS, with 304L-Cu SS’s corrosion current density being four times lower than 304SS’s due to copper’s bactericidal effects [121,122]. Similarly, HNS-Cu and 2205-Cu DSS yielded comparable outcomes [123,124]. Recently, microbial corrosion researchers have proposed enhancing the antimicrobial corrosion resistance of metal materials by adjusting their metallographic ratio or grain size at the microstructural level [125]. For example, acicular ferrite pipeline steel is superior to traditional X80 steel in terms of antibacterial activity against SRB and *Pseudomonas aeruginosa*, as well as pitting corrosion resistance [126].

### 5.4. Biological Protection

Microorganisms can play a dual role in the environment due to the complexity of their metabolisms. Compared to conventional anticorrosion techniques, microbial corrosion inhibition is more efficient and environmentally friendly. Some corrosive microorganisms can protect metals by altering environments beneath the biofilm. In the actual marine environment, the mechanism of microbial corrosion inhibition is more complex than traditional strategies. The main mechanisms of microbiologically influenced corrosion inhibition (MICI) comprise corrosion inhibitor secretion, biofilm, corrosion product shielding effects, alterations in the local micro-environment, and modifications in anodic and cathodic processes [127].

**Antimicrobial active substance**. Microorganisms have been found to secrete a range of compounds that can inhibit corrosion of metals. These include amino acids such as poly-aspartic acid and γ-polyglutamic acid, biosurfactants, proteases, alkaline phosphatases, and carbonic anhydrases [128,129,130]. Proteases, for example, can affect the pH of the environment and promote the sedimentation of ions, which, in turn, helps to protect metals [131]. Studies have shown that *Bacillus* sp. and *Pseudomonas fluorescens* secrete surfactants that can slow down corrosion on stainless steel 304 and carbon steel ST37 [131,132]. Additionally, the acidic extracellular secretions on the surface of microorganisms are rich in cations that can adsorb and form a natural barrier layer on the metal surface, thus reducing the corrosion rate of metals [131,132]. However, the potential for antimicrobial agents to dissolve into the environment remains a topic of discussion.

**EPS protection**. Biofilms are formed by the adhesion and aggregation of microorganisms, including bacteria and extracellular polymers (EPS). These biofilms exhibit specific strength and viscosity and demonstrate several characteristics. Qu et al. (2015) found that a nutrient-rich simulated seawater-based medium caused Bacillus subtilis to form biofilms on the surface of cold-rolled steel [133]. In the presence of *Bacillus subtilis*, a decrease in open circuit potential (OCP) was observed when compared to sterile artificial seawater medium, and corrosion inhibition was evident after the formation of the biofilm. The interactions between various types of bacteria in biofilms and metals are complex and require further research. EPS is a significant component of biofilm and contains polysaccharides with carboxyl groups that contain C–O and C=O bonds [134]. These groups can be complexed with iron and other metal ions to form a dense protective layer. Recent research indicates that specific microorganisms can inhibit corrosion by forming biofilms in the actual marine environment [135]. *Bacillus subtilis* has been identified as the main strain attached to the natural biofilm on the surface of low alloy steel [136]. A pure culture of *Bacillus subtilis* was isolated and purified, and its effect on the corrosion of low-alloy steel was studied [135]. *Bacillus subtilis* forms a uniform and hydrophobic biofilm composed mainly of polysaccharide and starch fiber, which can withstand unfavorable physical and chemical conditions and provide the biofilm with structural stability. The scanning vibrating electrode technique (SVET) results show that the current distribution on the sample surface is uniform, indicating that *Bacillus subtilis* has formed a uniform film [135].

## 6. Conclusions

Developing novel materials and ensuring secure operations in the maritime industry requires a comprehensive understanding of MIC. While recent research has primarily focused on MIC by a single microbial strain, it is crucial to acknowledge that microorganisms interact with each other in real ecosystems. Therefore, it is essential to replicate conditions that closely resemble real-world situations. MIC is affected by various factors, such as microbial metabolism, community distribution, attachment, and evolution, which are challenging to quantify statistically. To develop effective strategies with which to inhibit microbial corrosion, mechanistic and empirical models of mixed microbial corrosion should be created, incorporating the evolution of dominant microbial community species and changes in microbial community structure.

Though effective, traditional anti-corrosion strategies, such as corrosion inhibitors and coatings, pose a risk of polluting the environment. MICI offers a promising and eco-friendly way for exploring novel corrosion prevention strategies. For instance, biomineralization and bacterial secretion substances can be combined to inhibit corrosion. Additionally, a new intelligent self-repairing anti-corrosion layer can be designed by utilizing biomineralization mechanisms. To ensure stable and lasting corrosion inhibition, other anti-corrosion methods such as eco-friendly corrosion inhibitors, green anti-corrosion coatings, novel antibacterial materials, and nanoparticles can also be integrated into MICI technology.

## Data Availability

No new data were created or analyzed in this study. Data sharing does not apply to this article.
